# Supplementary material for: Electric-field control of the nucleation and motion of isolated three-fold polar vertices
Source: Nat Commun. 2022 Oct 25;13:6340. doi: 10.1038/s41467-022-33973-8 (PMC9596422; doi:10.1038/s41467-022-33973-8)
Supplement: Supplementary file 1 — Supplementary Information [file 41467_2022_33973_MOESM1_ESM.pdf]

**Supplementary Information**

# Electric-field control of the nucleation and motion of isolated three-fold polar vertices

Mingqiang Li[1,2], Tiannan Yang[3], Pan Chen[4], Yongjun Wang[5], Ruixue Zhu[1], Xiaomei Li[1], Ruochen Shi[1], Heng-Jui Liu[6], Yen-Lin Huang[5], Xiumei Ma[1], Jingmin Zhang[1], Xuedong Bai[4,7,8], Long-Qing Chen[3], Ying-Hao Chu[5,9], Peng Gao[1,10,11,12*]

[1]Electron microscopy laboratory, and International Center for Quantum Materials, School of Physics, Peking University, Beijing 100871, China;
[2]Department of Materials Science and Engineering, University of Toronto, Toronto, ON M5S 3E4, Canada;
[3]Department of Materials Science and Engineering, The Pennsylvania State University, University Park, PA, 16802, United States;
[4]Beijing National Laboratory for Condensed Matter Physics, Institute of Physics, Chinese Academy of Sciences, Beijing 100190, China;
[5]Department of Materials Science and Engineering, National Yang Ming Chiao Tung University, Hsinchu, 30010, Taiwan, ROC;
[6]Department of Materials Science and Engineering, National Chung Hsing University, Taichung, 40227, Taiwan, ROC;
[7]School of Physical Sciences, University of Chinese Academy of Sciences, Beijing 100190, China.
[8]Songshan Lake Materials Laboratory, Dongguan, Guangdong 523808, China.
[9]Institute of Physics, Academia Sinica, Taipei 11529, Taiwan, ROC;
[10]Collaborative Innovation Centre of Quantum Matter, Beijing 100871, China;
[11]Interdisciplinary Institute of Light-Element Quantum Materials and Research Center for Light-Element Advanced Materials, Peking University, Beijing, 100871, China;
[12]Hefei National Laboratory, Hefei, 230088, China.

[*]Peng Gao.
Email: p-gao@pku.edu.cn

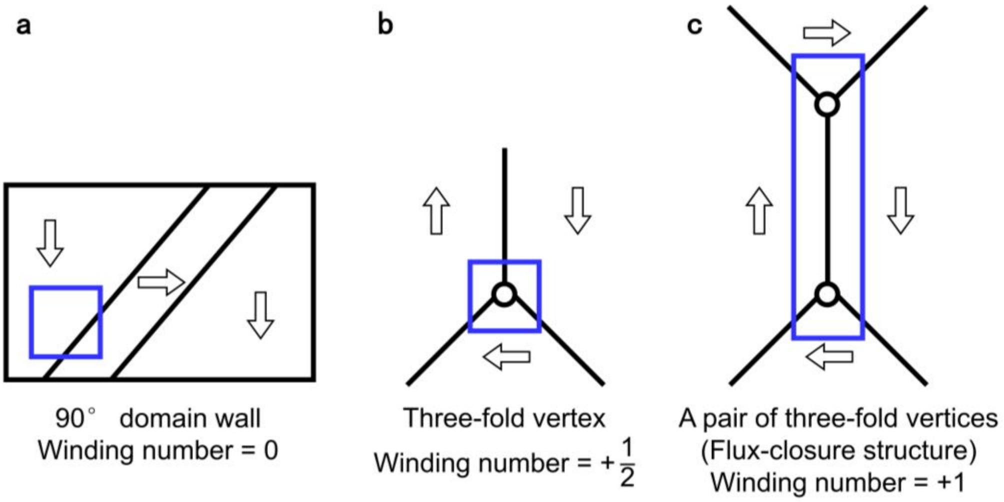


**Supplementary Figure. 1. Schematic diagrams of a 90° domain wall a, three-fold vertex b, and flux-closure polar structure c corresponding with their winding numbers.**

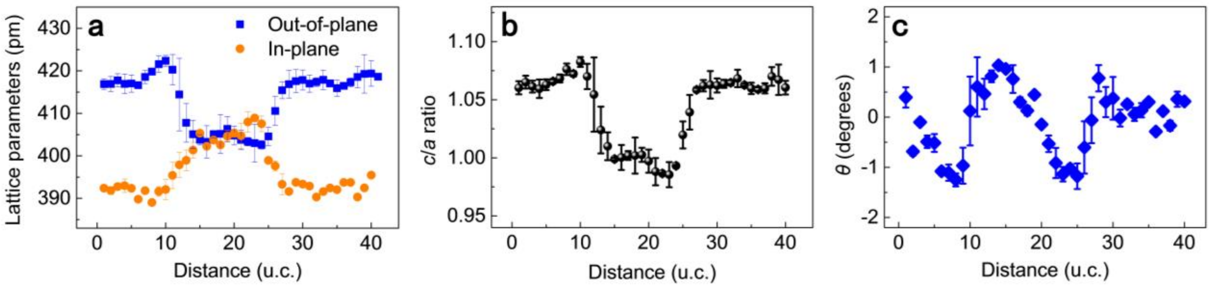


**Supplementary Figure 2. Lattice parameters of three-fold vertices.** Out-of-plane lattice parameters **a**, in-plane lattice parameters **b** and lattice rotation **c** of the three-fold vertex corresponding to marked regions in Fig. 1**d-f**, respectively.

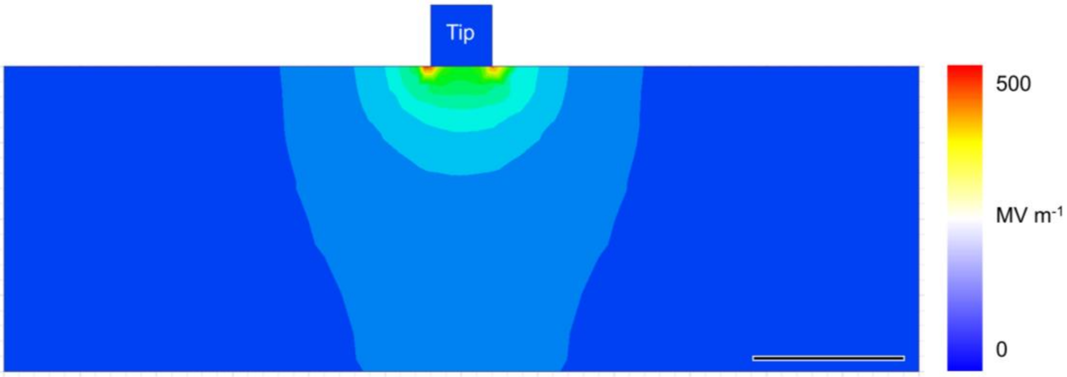


**Supplementary Figure 3. The distribution of electric field in $PbTiO_3$ films under -8 V.** The field distribution is modeled by Ansoft Maxwell software. The tip contact width is estimated to be 20 nm. Scale bar, 50 nm.

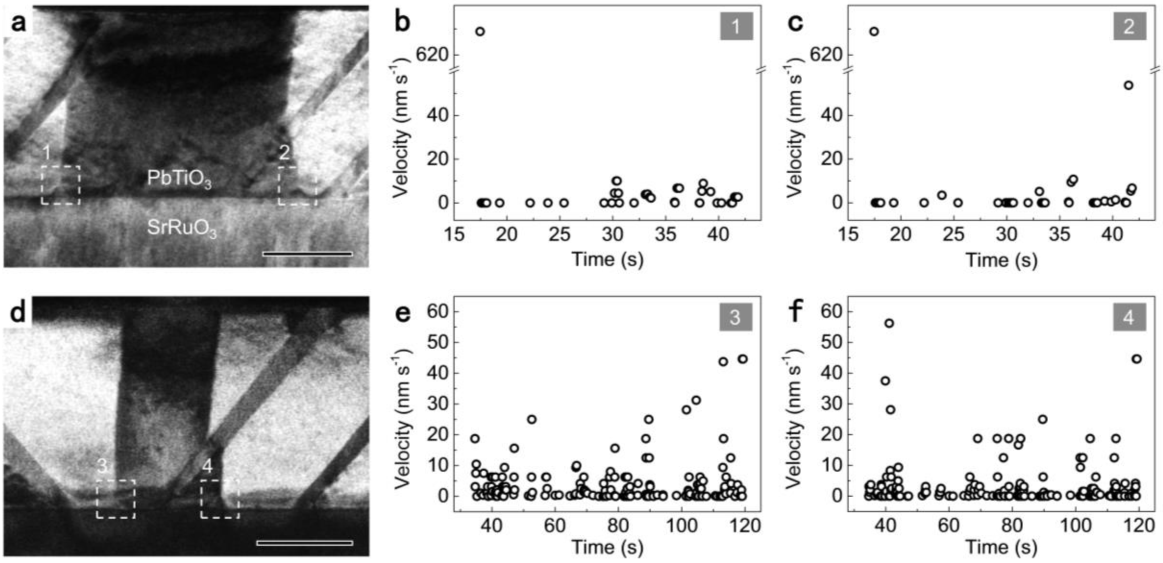


**Supplementary Figure 4. Measured velocities of isolated three-fold vertices under electric fields.** **a** TEM dark-field image of two three-fold vertices at the $PbTiO_3/SrRuO_3$ interface in Fig. 3. Scale bar, 50 nm. The corresponding velocities of these two three-fold vertices are shown in **b** and **c**, respectively. **d** TEM dark-field image of two three-fold vertices at the $PbTiO_3/SrRuO_3$ interface in Supplementary Fig. 4. Scale bar, 50 nm. The corresponding velocities of these two three-fold vertices are shown in **e** and **f**, respectively.

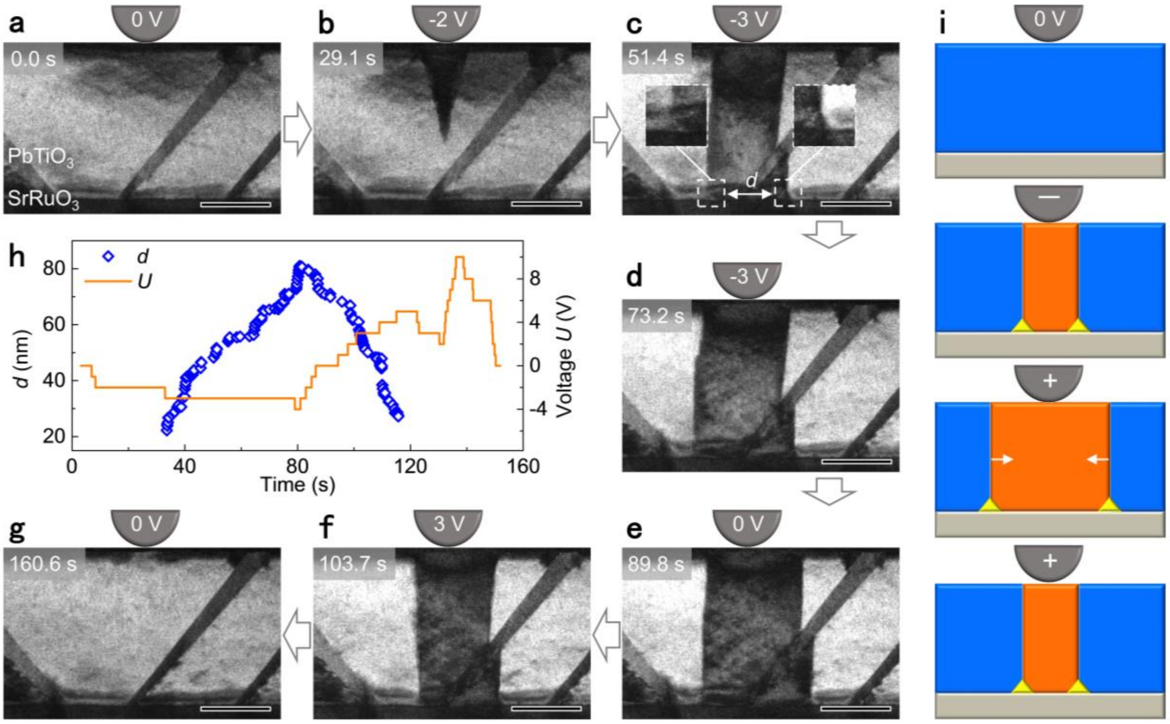

**Supplementary Figure 5. Electric-field control of isolated three-fold vertices at the $PbTiO_3/SrRuO_3$ interface. a-g** Chronological TEM dark-field image series illustrates nucleation and lateral motion of two three-fold vertices under applied electric fields. Scale bar, 50 nm. **h** Plots of applied voltage (orange line) and distance between two isolated three-fold vertices (blue diamond) as functions of time. **i** Schematic diagrams of the electric-field-driven motion of three-fold vertices. White arrows indicate the motion direction of domain walls.

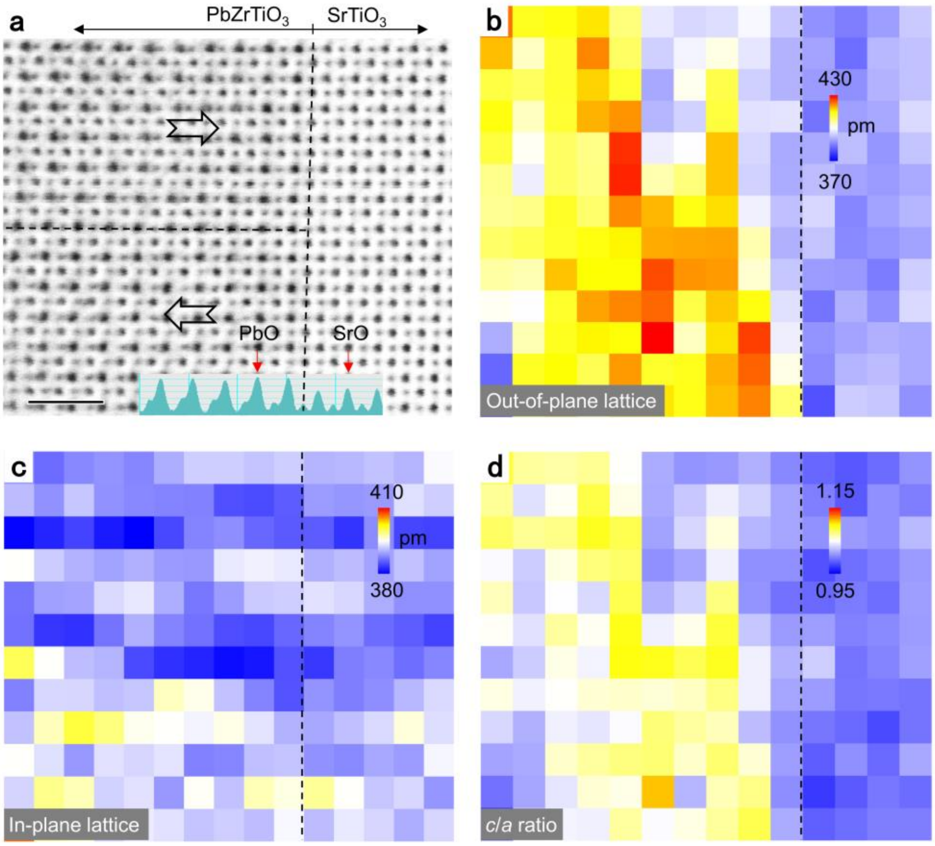


**Supplementary Figure 6. Atomic structure of 180° domain wall at the $PbZrTiO_3/SrTiO_3$ interfaces. a** An annular bright field (ABF) image of the $PbZrTiO_3/SrTiO_3$ interface with a 180° domain walls. Scale bar, 1 nm. Open arrows show the direction of polarization. The inset intensity profile indicated the location of the $PbZrTiO_3/SrTiO_3$ interface. Quantitatively atomic structure analysis showing the distribution of out-of-plane lattice parameters **b**, in-plane lattice parameters **c** and corresponding *c*/*a* ratio **d**. Dashed lines indicate the location of the $PbZrTiO_3/SrTiO_3$ interface.

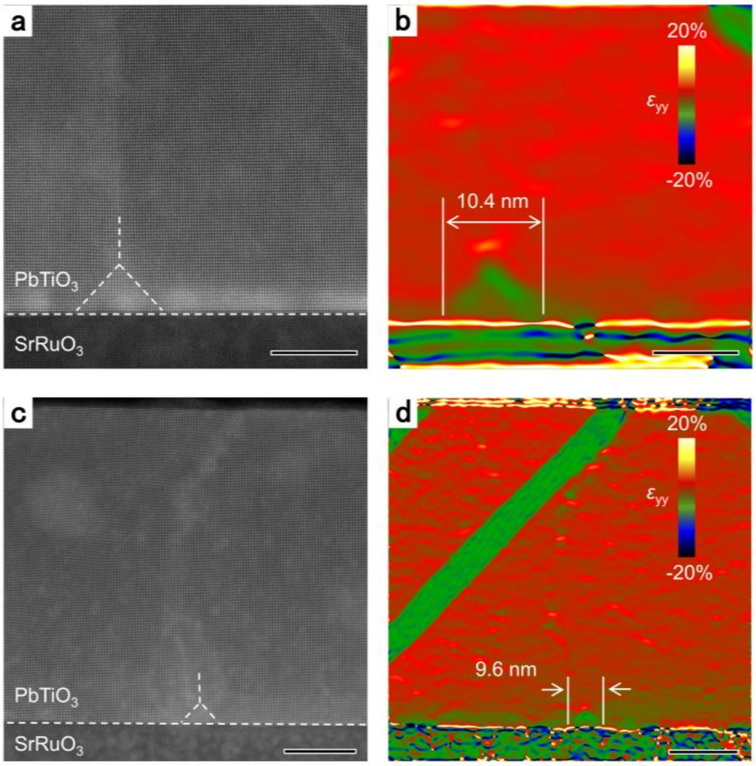


**Supplementary Figure 7. Strain distribution of three-fold vertices by GPA analysis. a** An atomically resolved HAADF-STEM image of an isolated three-fold vertex at the $PbTiO_3/SrRuO_3$ interface. Scale bar, 10 nm. **b** Corresponding out-of-plane strain distribution ($\varepsilon_{yy}$) from GPA analysis showing the strain field of the isolated three-fold vertex is localized, ~ 10 nm. **c** An atomically resolved HAADF-STEM image of an isolated three-fold vertex at the $PbTiO_3/SrRuO_3$ interface. Scale bar, 20 nm. **d** Corresponding out-of-plane strain distribution ($\varepsilon_{yy}$) from GPA analysis showing the strain field of the isolated three-fold vertex is localized, ~ 10 nm.

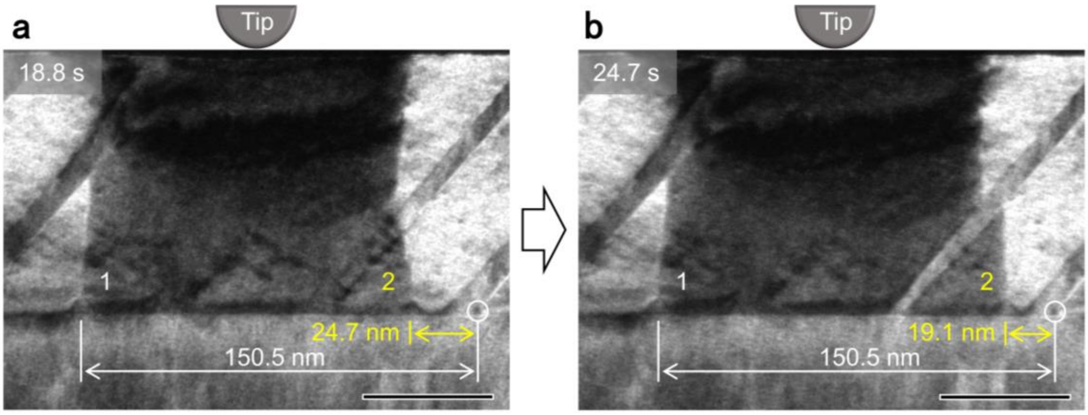

**Supplementary Figure 8. The independent motion of two isolated three-fold vertices under electric fields.** From **a** to **b**, three-fold vertex 2 moves to the right while three-fold vertex 1 is motionless. A white circle marks the reference of the measurement. Scale bar, 50 nm.

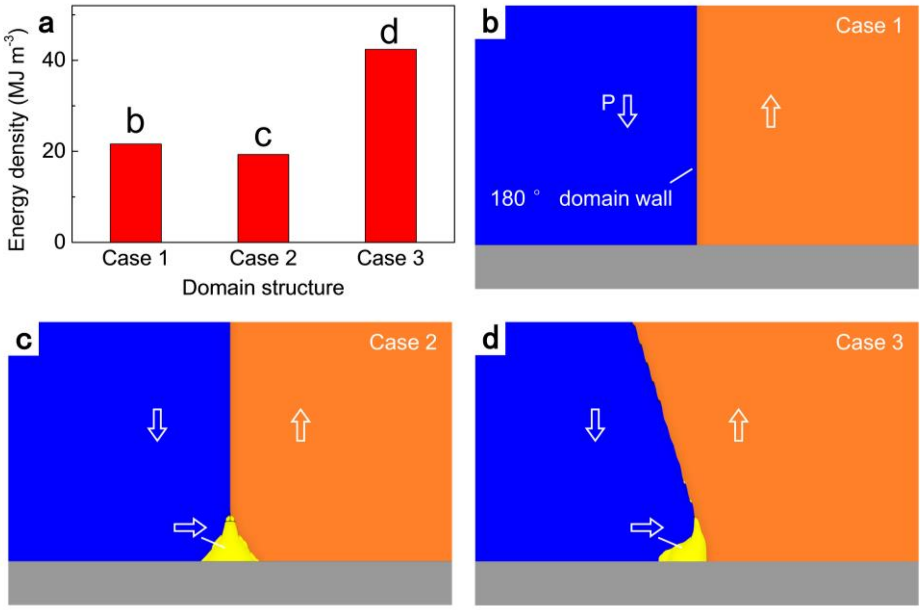


**Supplementary Figure 9. Phase-field simulation of the formation energy. a** Comparison of the formation energy of systems with different domain wall configurations from phase-field simulations, including a 180° domain wall without the formation of isolated three-fold vertices in **b**, a 180° domain wall with the formation of an isolated three-fold vertex in **c**, a tilted 180° domain wall with the formation of an isolated three-fold vertex in **d**.
