# Supplementary material for: Electric-field control of the nucleation and motion of isolated three-fold polar vertices
Source: Nat Commun. 2022 Oct 25;13:6340. doi: 10.1038/s41467-022-33973-8 (PMC9596422; doi:10.1038/s41467-022-33973-8)
Supplement: Supplementary file 2 — Description of Additional Supplementary Files [file 41467_2022_33973_MOESM2_ESM.pdf]

## Description of Additional Supplementary Files

**Supplementary Movie 1**. Electric-field-driven formation and motion of isolated three-fold vertices at the $PbTiO_3/SrRuO_3$ interface.

**Supplementary Movie 2**. Repeated experiment of the formation and motion of isolated three-fold vertices at the $PbTiO_3/SrRuO_3$ interface.
