# Supplementary material for: Electric-field control of the nucleation and motion of isolated three-fold polar vertices
Source: Nat Commun. 2022 Oct 25;13:6340. doi: 10.1038/s41467-022-33973-8 (PMC9596422; doi:10.1038/s41467-022-33973-8)
Supplement: Supplementary file 5 — Lasing Reporting Summary [file 41467_2022_33973_MOESM5_ESM.pdf]

nature research

Corresponding author(s): Peng Gao

Lasing Reporting Summary

Nature Research wishes to improve the reproducibility of the work that we publish. This form is intended for publication with all accepted papers reporting claims of lasing and provides structure for consistency and transparency in reporting. Some list items might not apply to an individual manuscript, but all fields must be completed for clarity.

For further information on Nature Research policies, including our data availability policy, see Authors & Referees.

ü Experimental design

Please check: are the following details reported in the manuscript?

1. Threshold

Plots of device output power versus pump power over a wide range of values indicating a clear threshold

- [ ] Yes
- [x] No

There were no lasing measurements. The laser was utilized to grow samples by a pulsed laser deposition system. We provided the wavelength, energy density and repeated rate of the laser in the Methods section of the manuscript.

2. Linewidth narrowing

Plots of spectral power density for the emission at pump powers below, around, and above the lasing threshold, indicating a clear linewidth narrowing at threshold

- [ ] Yes
- [x] No

There were no lasing spectral measurements.

Resolution of the spectrometer used to make spectral measurements

- [ ] Yes
- [x] No

There were no lasing spectral measurements.

3. Coherent emission

Measurements of the coherence and/or polarization of the emission

- [ ] Yes
- [x] No

No measurement is related to the coherence of the laser.

4. Beam spatial profile

Image and/or measurement of the spatial shape and profile of the emission, showing a well-defined beam above threshold

- [ ] Yes
- [x] No

Not necessary for growing samples by pulsed laser deposition method.

5. Operating conditions

Description of the laser and pumping conditions
Continuous-wave, pulsed, temperature of operation

- [x] Yes
- [ ] No

The repetition rate is 10 Hz as provided in the Methods section of the manuscript.

Threshold values provided as density values (e.g. W cm^{-2} or J cm^{-2}) taking into account the area of the device

- [x] Yes
- [ ] No

The energy density is about 2.5 mJ cm-2 as provided in the Methods section of the manuscript.

6. Alternative explanations

Reasoning as to why alternative explanations have been ruled out as responsible for the emission characteristics
e.g. amplified spontaneous, directional scattering; modification of fluorescence spectrum by the cavity

- [ ] Yes
- [x] No

There were no lasing measurements.

7. Theoretical analysis

Theoretical analysis that ensures that the experimental values measured are realistic and reasonable
e.g. laser threshold, linewidth, cavity gain-loss, efficiency

- [ ] Yes
- [x] No

There were no lasing measurements.

8. Statistics

Number of devices fabricated and tested

- [ ] Yes
- [x] No

There were no devices fabricated in this study. The laser is used to grow samples by a pulsed laser deposition system.

Statistical analysis of the device performance and lifetime (time to failure)

- [ ] Yes
- [x] No

There was no statistical analysis on devices.
